# Microplastics in Freshwater Ecosystems of India: Current Trends and Future Perspectives

**DOI:** 10.1021/acsomega.3c01214

**Published:** 2023-09-16

**Authors:** Kannaiyan Neelavannan, Indra Sekhar Sen

**Affiliations:** Department of Earth Sciences, Indian Institute of Technology Kanpur, Kanpur, UP 208016, India

## Abstract

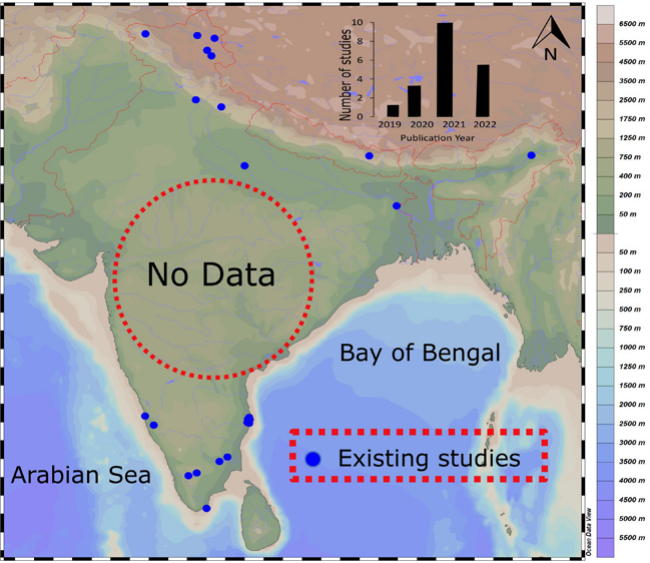

Microplastics (MPs)—i.e., plastic particles less than 5 mm in length—are becoming a growing environmental concern due to their potential ecotoxicological impacts on aquatic ecosystems. In India, MPs contamination is a significantly growing problem due to increased plastic production as well as its low rate of recycling. As a result, MPs research work in India has gained considerable attention in the last two decades. The objective of this study is to conduct a comprehensive review of the existing scientific literature on MPs in freshwater ecosystems (e.g., lakes and rivers) of India. A bibliographical search was used to conduct the literature review across a number of databases including ScienceDirect, Google Scholar, and ResearchGate. We found that in comparison to the marine ecosystem the source, transport, and fate of MPs in freshwater ecosystems of India are still underexplored, and we found only 18 relevant papers. This review work reveals that there is no standard procedure for separating MPs from water and sediment samples, and as a result, comparing the results was a challenging task. The larger MPs (>500 μm) in water and sediments were identified most commonly using the attenuated total reflection (ATR) Fourier Transform Infrared (FTIR) spectroscopy technique (ATR-FTIR), whereas smaller-sized MPs (<500 μm) were identified using FTIR fitted with a confocal microscope, also known as μ-FTIR imaging or chemical imaging. We found that white-colored fibers and fragments of polypropylene (PP), polyethylene terephthalate (PET), and polyethylene (PE) were the most common polymer types in the freshwater ecosystems of India. Although research on MPs in freshwater ecosystems of India has gained momentum over the past decade, the literature review reveals a limited understanding of the impact of MPs’ weathering patterns, the role of biofouling, and the role of water hyacinths on freshwater ecosystem services in India. Furthermore, the fluxes of MPs to the Indian oceans are not constrained, and atmospheric transport in high-altitude mountains, which have already been made fragile by climate change, has not been fully investigated. This study, therefore, calls for additional assessments of MPs in freshwater ecosystems—particularly in the central parts of India.

## 1 Introduction

Plastic pollution is a significant global problem due to the impact of plastic on the environment, and only a small portion of plastics are recycled.^1^ In 2018, global polymer production resulted in 359 million tons, but only 47.1% of plastic waste was properly disposed of through recycling, landfills, and energy recovery. The problem is projected to grow in the future as the plastic production rate is projected to double in the next two decades.^2^ While plastic is a significant discovery that has dramatically transformed our way of life, its nondegradable properties, as well as issues with recycling, pose great harm to the environment.^1^

Plastic contamination possesses a more serious concern in countries with a high population density such as India. Questions and concerns have been raised on the impact of plastic pollution in the freshwater ecosystems of India, in particular the rivers that directly or indirectly support a billion people in the Indian subcontinent.

India has one of the world’s largest river networks in the world,^3^ which includes 12 major, 7 medium, and many minor rivers and watercourses, with an estimated total length of around ∼16 × 10^4^ km.^4^ In addition to rivers, India also possesses a variety of aquatic environments, such as lakes, ponds, canals, estuaries, floodplains, coastal water bodies, and marine systems. India’s economic development is heavily reliant on its freshwater aquatic environment, which is essential for a range of activities such as agriculture, aquaculture, navigation, electricity generation, and various industrial and commercial operations.^6^ For example, India is the world’s second-largest producer of fisheries and aquaculture, with a fish production of 108 × 10^5^ tonnes in the fiscal year 2014–15. It also has the second-largest diversity of aquatic fish in Asia, officially recognizing 2319 fish species, of which 838 are freshwater species. It is worth mentioning that India currently contributes 5% of the world’s fish trade and 6.3% of the world’s total fish consumption,^7^ and the industry is a significant source of income for over 14.5 million people in the nation’s economically disadvantaged population.^8^ Therefore, any pollution threat to the country’s freshwater resources will impact the country’s economy, development, and growth.

The Indian freshwater ecosystems are heavily contaminated^9,10^ due to the discharge of untreated wastewater from various sources, including industries, urban areas, agricultural fields, along with urban runoff.^11,12^ Surface water and groundwater sources are contaminated by various pollutants such as organic, inorganic, and plastic pollutants.^13−16^ Previous studies have produced a rich body of information concerning organic and inorganic contaminants.

Plastics are mainly categorized based on their size. Plastic pollutants can be divided into nanoplastic (<1 μm), microplastic (MPs; <1 μm to >0.5m), mesoplastic (<5 to >25 mm), and macroplastic (>25 mm).^20^ Another way to categorize MPs is based on their origin, i.e., primary and secondary MPs. Primary MPs are plastic microbeads from cosmetics and other products that enter the aquatic environment directly from human activity. Secondary MPs are plastic fragments resulting from the breakdown of larger plastic debris that degrades through various processes such as physical, photodegradation, chemical, and biodegradation.^21^

MPs can directly or indirectly affect human health by acting as carriers of physical stressors or environmental toxins.^22^ Recent studies have discovered MPs in human breast milk,^23^ blood samples,^24^ and lungs,^25^ suggesting that they can be absorbed by human blood and lung tissue. An et al.^26^ reported that the nonspherical MPs are more toxic than spherical ones. Gray and Weinstein^27^ showed that MPs smaller than 50 μm are less harmful to shrimp than those larger than 50 μm. Among various polymer types, poly(vinyl chloride) (PVC) and polyurethane (PUR) have a particularly harmful effect on biota.^28^ In general, the toxicity of MPs is dependent on their size, shape, and polymer type.

MPs have been detected in both freshwater and marine environments around the world, with higher levels found in densely populated urban areas and their waters, including precipitation, sewage sludge, treated wastewater effluent, and drinking water.^29−37^ Recent research has shown that the number of scientific papers on MPs pollution in freshwater environments has been steadily increasing, with about 40% of recent studies focusing on freshwater.^38−40^ Studies suggest that the abundance of MPs in freshwater is comparable to or even higher than that in marine environments.^41,42^ MPs can enter freshwater environments through various pathways, including agricultural runoff, industrial effluents, fishing activities, tourism, atmospheric fallout, plastic waste dumping, stormwater discharge, road runoff, flooding events, wastewater treatment plant effluents, and domestic sewage.^43,53^

In India, MPs research work is mostly focused on coastal environments, coastal sediments,^46,47^ seawater,^48^ biota,^49^ sea salt,^50^ lakes,^30,33,51^ and fish.^49^ To the best of our knowledge, only a few studies of MPs in India’s freshwater systems have been conducted to date. The objective of this study is to conduct a comprehensive review of the existing scientific literature on MPs in the freshwater ecosystems of India to better understand the impacts of MPs pollution on ecosystem services associated with freshwater resources.

## 2 Plastics in Indian Perspective

It is estimated that 8 to 12 million tonnes of plastic debris enter the ocean every year due to the mishandling of plastic waste in aquatic areas,^17,19^ and India has significantly contributed to this problem due to its population growth, urbanization, and industrialization.^17^ According to a study by the Central Pollution Control Board, Government of India (CITE), India generated 3.3 million metric tonnes of plastic waste in 2018–2019, and a significant amount of plastic waste was disposed of in open landfills.^18^

The issue of plastic pollution is an increasingly important problem that is well recognized in India, and numerous efforts are underway to reduce plastic footprints. For example, India was the first nation to ban single-use plastics on ships in 2019, highlighting its early awareness of the issue. The state of Sikkim first introduced a ban on single-use plastics in 1998, with other Indian states following suit. By 2022, all of India was set to prohibit the usage of single-use plastics.^44,45^ We mention that the plastics industry was established in India during the 1950s, but it was not prioritized by the government until the 1970s (All India Plastic Manufacturers’ Association, 2019). In recent decades, however, the industry has become a significant contributor to the Indian economy, with a multiplier effect on various sectors (Federation of Indian Chambers of Commerce and Industry, 2016). Despite its growth potential, the plastics industry in India still faces multiple challenges, including the need for proper waste management and concerns about negative environmental impacts.

## 3 Methodology

### 3.1 Study Area

India possesses one of the world’s most extensive river networks, with diverse water impoundments and rivers spread across a wide range of climates ranging from tropical climates in the south to mountainous climates in the north.^4^ Additionally, India has a vast coastline of approximately 7,500 km stretching along the Arabian Sea in the west and the Bay of Bengal in the east, with 13 coastal states and union territories (including islands).^21^ The country’s Exclusive Economic Zone (EEZ) spans over 2.5 million km^2^ with a large shelf area of 0.13 million km^2^.^21^ The Indian coast also harbors an incredible range of habitats, including seagrass beds, wetlands, mangrove swamps, mudflats, coral reefs, sand dunes, and rocky and sandy shorelines,^21^ and is home to the second-largest diversity of aquatic fish in Asia.^5^

### 3.2 Data Collection

**Figure 1:**
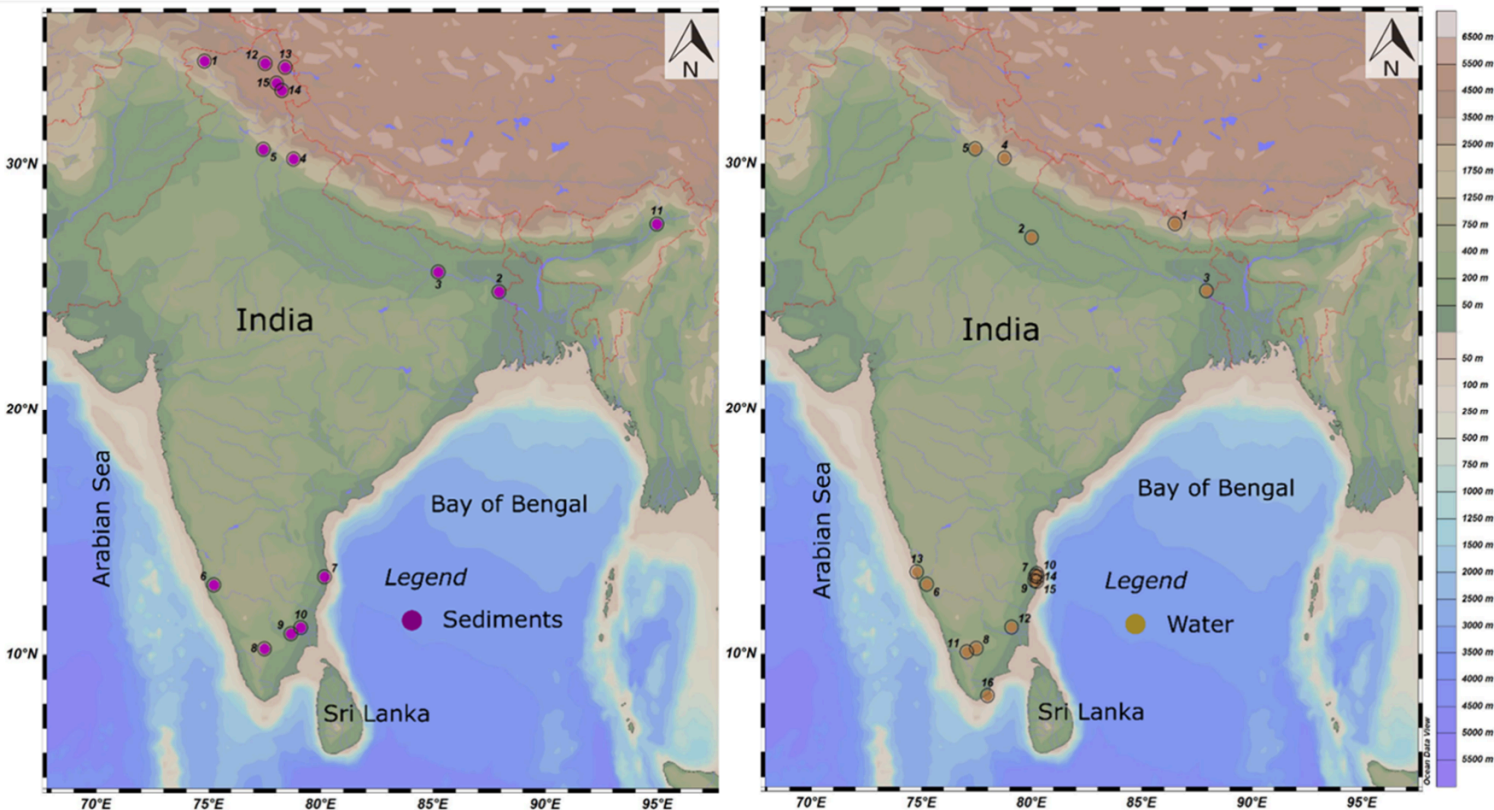
Map showing the study of MPs in freshwater environments in India. Sediment: 1. Neelavannan et al.^33^; 2. Singh et al.^57^; 3. Sarkar et al.^61^; 4. Chauhan et al.^58^; 5. Ajay et al.^63^; 6. Amrutha and Warrier^31^; 7. Gopinath et al.^30^; 8. Laju et al.^59^; 9. Maheswaran et al.^65^; 10. Bharath et al.^60^; 11, 12. Tsering et al.^18^; 13, 14, 15. Tsering et al.^62^ Water; 1. Napper et al.^70^; 2. Napper et al.^52^; 3. Singh et al.^57^; 4. Chauhan et al.^58^; 5. Ajay et al.^63^; 6. Amrutha and Warrier^31^; 7. Gopinath et al.^30^; 8. Laju et al.^59^; 9, 10, 11. Lechthaler et al.^32^; 12. Bharath et al.^68^; 13. Warrier et al.^51^; 14, 15. Bharath et al.^60^; 16. Selvam et al.^66^

A bibliographical search was used to conduct the literature review across a number of databases, including ScienceDirect, Google Scholar, and ResearchGate. Research publications (published up until June 30, 2022) were identified. The search terms were “Plastic Pollution”, “Plastic Debris”, “Microplastics”, “India”, “Freshwater”, “Lake”, “River”, “Sediment”, “Water”, and “Biota”. In terms of the publication year, there were no limitations. The search results were carefully analyzed, and only 18 studies that are relevant to the freshwater ecosystems of India were considered (Figure 1).

## 4 Sample Collection Methods

Despite the fact that research on MPs has been ongoing for almost two decades, there is still no standardization of methods for sample collection, pretreatment of collected samples, and identification and quantification of MPs.^54,55^ The sampling methods employed for the collection of data on MPs in water and sediment in India are outlined in Table 1. However, the presence of inconsistencies in analytical protocols poses a challenge to comparing results, which may be due to variations in sampling methodologies and pretreatment extraction techniques.^56^

**Table 1:** MPs Studies in Freshwater Ecosystems of India

Sediment	Location	Sample type	Size	Shape	Polymer type	Abundance	Extraction method	Detection method	Ref
1	Lower Ganga River	River sediment	1–5 mm	Films, foams, fragments, and filaments	PVC, PP, CP, PS, PE, poly(butadiene:acrylonitrile), polyvinyl chloride:ethylene, polyvinyltoluene:butadiene, polyethylene propylene, poly(trimellitic amide imide)	17–36 items/kg of dry weight (dw)	Sieving; treated with H_2_O_2_; density separation using NaCl; filtration through 47 mm glass filter	Microscope and ATR-FTIR	Singh et al. 2021^57^
2	Lower Ganga River, Eastern India	River sediment	63–100 mm	Fibers, monofilaments, films, fragments, foams, and beads	PET, PE, PP, PS	Meso and micro; 0.68–148.31 ng/g and 11.48–63.79 ng/g	Sieving; density separation using NaCl; filtration through 0.7 μm glass microfiber filter	Microscope and ATR-FTIR	Sarkar et al. 2019^61^
3	Alaknanda River, Uttarakhand	River sediment	1–5 mm	Fibers, fragments, films, and pellets	PT, HDPE, PVC, LDPE, PP, PS	389 MP particles	Sieving, treated with H_2_O_2_; density separation using NaCl; filtration through 5 μm membrane filter	Microscope, SEM, and EDS	Chauhan et al. 2021^58^
4	Netravathi River, Southern India	River sediment	0.3–5 mm	Fragments, fibers, and films	PE, PET, PP	9.44–253.27 items/kg dw	Dried in a hot-air oven at 90 °C overnight; sieving; treated with (NaPO_3_)_6_, H_2_O_2_; density separation using ZnCl_2_; filtration through 1 mm and 3 mm at the top and bottom	Microscope, ATR-FTIR, and SEM with EDS	Amrutha and Warrier 2020^31^
5	Brahmaputra River	River sediment	150 μm–5 mm	Fragments, fibers, and beads	PE, PP, PA, PTFE, PVC, PS	20–24 MP/kg dw	Sieving; treated with H_2_O_2_; density separation using sodium tungstate dihydrate (Na_2_WO_4_·2H_2_O); filtration through silver membrane filter (25 mm, pore size 0.5 μm, Sterlitech)	Microscope, μFTIR, and SEM	Tsering et al. 2021^18^
6	Indus River	River sediment	150 μm–5 mm	Fragments and fibers	PE, PP, PA, PS	60–340 MP/kg dw			
7	Kaveri River	River sediment	0.1–5 mm	Films, fibers, fragments, and foams	PA, PE, PET, PS, PP, PEG	1 to 699 ± 66.00 items/kg	Sieving; treated with H_2_O_2_; density separation using ZnCl_2_; filtration through filter membrane	Microscope, ATR-FTIR, and SEM-EDS	Maheswaran et al. 2022^65^
8	Anchar Lake, NW Himalaya	Lake sediment	0.3–5 mm	Fragments, pellets, and fibers	PS, PP, PA, PVC	233 to 1533 particles/kg	Sieving; treated with H_2_O_2_; density separation using NaCl; filtration through 0.45 μm cellulose nitrate	Microscope and ATR-FTIR	Neelavannan et al. 2022^33^
9	Red Hills Lake	Lake sediment	0.3–5 mm	Fibers, fragments, films, and pellets	PE, PP, HDPE, LDPE	Mean 27 particles/kg	Sieving; density separation using NaCl; filtration through Whatman GF/A filter paper (25 mm)	Microscope, ATR-FTIR, and SEM with EDS	Gopinath et al. 2020^30^
10	Kodaikkanal Lake	Lake sediment	0.25–5 mm	Fragments, films, foams, and fibers	PE, PP, PS, PET, polyvinyl alcohol	Mean 28.31 ± 5.29 items/kg	Sieving; treated with H_2_O_2_; density separation using ZnCl_2_; filtration through 0.8 μm cellulose nitrate filter paper	Microscope, ATR-FTIR, and SEM with EDAX	Laju et al. 2022^59^
11	Kodaikkanal Lake	Lake sediment (core)	0.25–5 mm	Fibers, fragments, films, foams, and pellets	PE, PP, PET, PS, PVA, PEU, CP	Mean 25.91 ± 7.11 items/kg			
12	Veeranam Lake	Lake sediment	0.3–5 mm	—	PVC, PE, PP, PS, NY	309 items/kg	Wet-sieving; treated with H_2_O_2_; density separation using ZnCl_2_	Microscope and ATR-FTIR	Bharath et al. 2021^60^
13	Renuka Lake	Lake sediment	0.2 μm–5 mm	Fragments, fibers films, and foams	PP, PS, PE	180 ± 143 particles/kg dw	Oven dried at a temperature of 50 °C; sieving; treated with Fentons reagent (WPO); density separation using NaCl, ZnCl_2_ filtration	Microscope, ATR-FTIR, and Raman spectroscopy	Ajay et al. 2021^63^
14	Pangong Lake	Lake sediment	100–5000 μm	Fragments and fibers	PE, PP, PS, PA, PET, POM, PMMA	160–1000 MP/kg dw	Na_2_WO_24_·2H_2_O; enzymes and fentons	Raman spectroscopy	Tsering et al. 2022^62^
15	Tsomoriri Lake	Lake sediment				960–3800 MP/kg dw			
16	Tsokar Lake	Lake sediment				160–1000 MP/kg dw			

Water	Location	Sample type	Size	Shape	Polymer type	Abundance	Extraction method	Detection method	Ref
1	Ganges River, India	River water	>300 mm	Fiber and fragment	Rayon, acrylic, PET, PVC, PS, nylon	Average 0.038 MP L^–1^, 140 MP, 3600 L	Pumped from 0.5 m below the river surface and immediately filtered through 330 mm nylon mesh	Microscope and ATR-FTIR	Napper et al., 2021^52^
2	Lower Ganga River	River water	1–5 mm	Film, fragment, pellets, films, and foam	PVC, PE, CP, PP, polytrimellitic amide imide, polytetrafluoroethene:propene, polystyrene, polyethylene vinyl chloride, polyacrylonitrile vinyl chloride	380–684 items/1000 m^3^, mean 466 items/1000 m^3^	Sieving; treated with H_2_O_2_; density separation using NaCl; filtration through 47 mm glass filters	Microscope and ATR-FTIR	Singh et al., 2021^57^
3	Alaknanda River, Uttarakhand	River water	1–5 mm	Fibers, fragments, pellets, films, and foam	PT, HDPE, PVC, LDPE, PP, PS	566 MPs	Sieving; treated with H_2_O_2_; density separation using NaCl; filtration through 5 μm membrane filters	Microscope, SEM, and EDS	Chauhan et al., 2021^58^
4	Netravathi River, Southern India	River water	0.3–5 mm	Fibers, films, foams, and pellets	PE, PET, PP, PVC	56 to 2328 pieces/m^3^, 288 pieces/m^3^	Dried in a hot-air oven at 75 °C for 24 h; Sieving; treted with H_2_O_2_(WPO); density separation using ZnCl_2_; filtration through 1 mm and 0.3 mm at the top and bottom	Microscope, ATR-FTIR, and SEM with EDS	Amrutha and Warrier, 2020^31^
5	Adyar River	River water	335 μm–5 mm	Fibers, films, fragments, and pellets	PE, PP, PS	Mean 330 items/m^3^	Sieving; oil separation using canola oil; filtration through Whatman 0.7 μm pore size, 47 mm	Microscope and ATR-FTIR	Lechthaler et al., 2021^32^
6	Kosasthalaiyar River	River water				Mean 670 items/m^3^			
7	Muthirappuzhayar River	River water				Mean 200 items/m^3^			
8	Red Hills Lake	Lake water	0.3–5 mm	Fibers, fragments, films, and pellets	PE, PP, HDPE, LDPE	Mean 5.9 particles/L	Sieving; density separation using NaCl; filtration through Whatman GF/A filter paper (25 mm)	Microscope, ATR-FTIR, and SEM with EDS	Gopinath et al., 2020^30^
9	Kodaikanal Lake	Lake water	0.25–5 mm	Fibers, fragments, foams and films	PE, PP, PS, PET	Mean 24.42 ± 3.22 items/L	Sieving; treated with H_2_O_2_; density separation using ZnCl_2_; filtration through 0.8 μm cellulose nitrate filter paper	Microscope, ATR-FTIR, and SEM with EDAX	Laju et al., 2022^59^
10	Veeranam Lake	Lake water	-	-	PVC, PE, PP, PS, NY	28 items/km^2^	Sieving; treated with H_2_O_2_; filtration through 0.45 μm Whatman glass microfiber filter paper	Microscope and ATR-FTIR	Bharath et al., 2021^60^
11	Manipal Lake	Lake water	0.3–5 mm	Fibers, films, pellets, and fragments	PET, CL	MS; 0.423 particles/L, PMS; 0.117 particles/L	Sieving; treated with H_2_O_2_ (WPO); density separation using ZnCl_2_; filtration through 1mm and 0.3 mm at the top and bottom	Microscope, ATR-FTIR, and SEM with EDS	Warrier et al., 2022^51^
12	Renuka Lake	Lake water	0.2 μm–5 mm	Fragment, fiber, pellet, film, and foam	PP, PS, PE	21 ± 13 particles/L	Filtered using glass fiber filter, 47 mm and pore size of 0.2 μm	Microscope, ATR-FTIR, and Raman spectroscopy	Ajay et al., 2021^63^
13	Mount Everest, various locations	Snow, stream water	36–3800 mm	Fiber and fragment	PE, PP acrylic, nylon	3–119 MP L^–1^ and 0–2 MP L^–1^	-	Microscope and ATR-FTIR	Napper et al., 2020^70^
14	Kodungaiyur, Chennai	Groundwater	-	-	-	3–23 items/L	Filtered with a 0.45 mm Whatman cellulose nitrate filter paper	Microscope, ATR-FTIR, and SEM with EDS	Bharath et al., 2021^68^
15	Perungudi, Chennai	Groundwater				7–80 items/L			
16	Tiruchendur groundwater	Bore and well water	2–5 mm	Fiber, foam pellet, film, fragment	PA, PE, PE	Mean 4.2 particles/L	Sieving; treated with H_2_O_2_; filtration through microline filter paper	Microscope, ATR-FTIR, μFTIR, and atomic force	Selvam et al., 2021^66^

### 4.1 Water

Thirteen articles have investigated the presence of MPs in various sources of water, including lakes (5), rivers (7), groundwater (3), and glaciers (snow and stream; 1). The samples were collected from depths ranging from 20 cm to 3–5 m using plankton nets with different mesh sizes (20, 100, 120, 300, 333, and 335 m).^30,57−60^ Some studies used a stainless-steel bucket and sieved stainless-steel mesh,^31,51^ while Napper et al.^52^ used a hand-operated bilge pump with a targeted MPs size range (>300 μm) to collect river surface water from a depth of 0.5 m from the Ganga River, which was then filtered through a 330 μm nylon mesh. The amount of water sampled was determined by attaching a flow meter to the manta trawl net. However, some studies did not specify whether a flow meter was used, the net speed, or the hours the net was towed. The accuracy of the measurement could be affected if the net was not completely submerged or blocked by debris, or sampling from the boat’s back or windward side could also impact quantification.^21^ Different types, sizes, and vessels with different speeds were used for sample collection. The concentration of MPs in water samples was expressed in various units, such as items/km^2^, items/m^3^, items/1000 m^3^, items/L, and particles/L.

### 4.2 Sediment

Thirteen studies focused on exploring the occurrence and distribution of MPs in the freshwater environment of India, specifically in lakes (8) and rivers (7). To collect MPs in sediment samples, stainless steel spoons/scoops and van-Veengrab samplers were most commonly used. Van-Veen grab samplers were also employed to obtain samples from the bottom surface or underwater, while shore samples (from lakes and rivers) were collected using stainless steel spoons/scoops with sampling depths ranging from 0 to 6 cm. Laju et al.^59^ collected core sediments to analyze the vertical distribution of MPs from India, which included 16 studies. The sampling units used for MPs were reported as particles/kg,^33^ particles/kg of dw,^57^ and items/kg,^31^ and some studies reported MP particles.^58^

## 5 MPs Separation Methods

All of the studies that have been published on MPs involve the separation of MPs from bulk sediments and water samples that were reduced in volume. Most studies attempted to perform density separation, although some relied on filtration or sieving of the sample before sorting, either through visual observation or under magnification. The differences in processing methods among the studies suggest that there is no established standard procedure for isolating MPs from environmental samples.

### 5.1 Water

In all 13 studies, the collected water samples were sieved or filtered to select the desired size. Digestion was conducted to remove organic matter using 30% H_2_O_2_, and MPs were extracted from water samples using density separation methods with NaCl^30,57,58^ and ZnCl_2_.^31,51^ Lechthaler et al.^32^ used canola oil for density separation to extract MPs from river water. Napper et al.^52^ used a hand-operated bilge pump to filter the water samples in the field, and the filters were placed in clean polypropylene (PP) bags for microscope and spectroscopy studies.

In order to understand PP bag contamination on the filters, the blanks for the filters were placed in PP bags. After digestion and density separation, the supernatant was filtered through filter papers of different mesh sizes, such as 0.2 μm, 0.45 μm, and 0.8 μm. The filter papers were then placed in Petri dishes and dried.

### 5.2 Sediment

In most cases, sediment samples were sieved using various mesh sizes, such as 10 mm,^61^ 5 mm,^31,58,59,61^ 2 mm,^30,33,60^ 1 mm,^30,33,60^ 0.3 mm,^30,33,60^ 850 μm,^61^ and 63 μm.^61^ With the exception of Tsering et al.,^62^ most studies dissolved organic matter by digesting with 30% H_2_O_2_ before density separation. MPs were extracted from sediments using the density separation method, utilizing NaCl in six studies, ZnCl_2_ in five studies, and Na_2_WO_4_·2H_2_O in two studies. After digestion and density separation, the supernatant fraction was filtered using filter paper with various mesh sizes, such as 0.2 μm,^63^ 0.45 μm,^30,33,60^ 0.7 μm,^61^ 5 μm,^18,58^ and 0.8 μm.^59^ Amrutha and Warrier^31^ used 1 mm and 0.3 mm sieves to separate the supernatant into two fractions, which were then transferred to two watch glasses and dried for further examination under a microscope and spectroscopic studies.

## 6 Identification, Quantification, and Confirmation of MPs

### 6.1 Visual Inspection

In all the reviewed studies, the most common method used for quantifying MPs in the freshwater environment of India was visual inspection,^64^ either with the naked eye or using a microscope/stereoscope. While larger MPs could be separated directly, smaller MPs required additional examination under a microscope. MPs were identified visually based on their brightness, homogeneous color, and absence of cellular features. Some researchers have employed visual identification along with hot-needle testing to confirm the presence of plastic (as opposed to organic or inorganic substances). Visual counting of MPs can be a time-consuming approach; it may also result in substantial overestimation or underestimation of plastic content depending on the size distribution of plastics and the possibility of mistaking nonplastic particles for plastic.

### 6.2 Fourier Transform Infrared Spectroscopy

The most commonly used technique for identifying and quantifying MPs is Fourier transform infrared (FTIR) spectroscopy. FTIR spectroscopy has a long history of use in investigating and characterizing MPs, providing an opportunity for precise identification of polymer types based on the characteristic fingerprint spectra of molecular vibrations. Among the 18 articles reviewed, the FTIR technique was employed in about 90% of the studies to determine the types of MPs polymers present in various environmental media (Table 1). The most popular FTIR spectral range in the MPs study is the mid-infrared region (400–4000 cm^–1^). The most common FTIR spectroscopy modes are transmission and attenuated total reflection (ATR). Larger MPs (>500 μm) in water and sediments were identified using the ATR-FTIR technique.^30−33,57,59−61,63,65^ The polymer types of smaller-sized MPs (<500 μm) in water^66^ and sediment^18^ were identified using FTIR fitted with a confocal microscope, also known as μ-FTIR imaging or chemical imaging. In addition to the characterization and identification of MPs, the weathering pattern or aging of MPs was also studied by utilizing the FTIR method using carbonyl index values. However, among the reviewed articles, the investigation of MPs has mostly ignored FTIR spectral preprocessing and chemometric methods.

FTIR and Raman microscopy are not advisable to scan the entire filters or quantify each individual particle, as it is time-consuming. For example, Raman measurements might take months or even years to complete on a single filter paper. The measurement timings in both FTIR and Raman microscopy surpass any practical time period for normal analysis, even with fully automated techniques. The area of the generated microscopic images using FTIR is 4 mm^2^, which represents only 0.82% of the overall filter area. A 4 mm^2^ filter area measurement can take anywhere between 30 and 120 min, which depends on how many particles are found. It takes a minimum of 48 h to measure the complete filter. Consequently, a general reduction in the covered filter surface is needed. In order to achieve results that are representative of the entire filter paper, a filter area larger than 0.82% must be explored, which necessitates averaging a multitude of images before extrapolating to the complete filter area.^85^ The generation of representative data sets can be done in a variety of ways, such as by calculating the filter area or total particle numbers.^84^ For both methods to be representative of the sample entity, there must be a large enough number of particles or a filter area. So, a template that covered between 8 and 20% of the entire filter area was created. Huppertsberg and Knepper.^85^ developed the scheme of the template, and details are shown in this publication by Huppertsberg and Knepper.^85^ A helical path across the filter can accommodate up to 21 tiny images. As the results of all microscopic photos are averaged before being extrapolated to the entire filter area, the location of the separate microscopic images was chosen to avoid misleading quantification owing to local particle hotspots.

Although FTIR is a promising method for identifying different types of MPs polymers, it has several limitations, including: (i) FTIR spectra for MPs obtained from various modes are not the same, (ii) a substrate is necessary to keep particles in place during spectrum collecting, but spectral interference caused by adding a substrate filter has not been properly addressed, (iii) the FTIR technique cannot analyze MPs smaller than 10 μm, (iv) before identifying MPs, it is essential to study how chemical degradation affects the FTIR spectral bands of plastics, (v) small, irregularly shaped MPs would generate unintelligible FTIR spectra due to refractive error, and (vi) broad peaks over 3000 cm^–1^ are produced by water content, which makes FTIR highly sensitive. Therefore, sample preparation is required before measurement.

### 6.3 Raman Spectroscopy

The Raman technique, including both microscopy and spectroscopy, is widely used for identifying MPs polymer types. Two studies used Raman techniques to identify MPs in sediment and water. Extracted MPs from Renuka Lake water and sediments were obtained with spectra between 500 and 3200 cm^–1^, and MPs were observed and analyzed under the 785 nm laser, with a 1200 grating and a 10 s exposure duration.^63^ Tsering et al.^62^ used the μ-Raman imaging microscope technique to characterize MPs polymer types from Indian Himalayan Lake sediments. The effective filtering area of each sample was generated as a 110 μm thick terrain mosaic by adding 9 μm at a time with a 10× objective. Particles larger than 100 μm were taken for polymer characterization and were scanned by using a 785 nm laser with 10 mW power, 0.1 s exposure duration, and 10 scans. The μ-Raman spectra between 600 and 1800 cm^–1^ were obtained for polymer identification. Raman spectroscopy can detect MPs as small as 1 μm and simultaneously assess the size distribution, morphological parameters, and particle numbers. Raman spectroscopy has a greater lateral resolution (1 μm vs 20 μm) than FTIR spectroscopy, greater spectral coverage, a highly distinct fingerprint spectrum, and less water interference. However, Raman spectroscopy’s disadvantage is the poor intensity of Raman scattering, which requires long acquisition times to obtain a good signal-to-noise ratio. To characterize MPs that are smaller than 20 μm, Raman microscopy is utilized, although it has weak signal limitations that can be addressed by extending measurement times and reducing fluorescence interference, which depend on the material properties such as biofouling, color, and degradation. Since the Raman spectra of weathered plastics are prone to change and there is no dedicated Raman database of weathered plastics, it is essential to develop a spectral database of weathered plastics and use it to identify unknown MPs in environmental samples.^67^

### 6.4 Scanning Electron Microscope and Energy Dispersive X-ray Spectrometer

The morphology, aging, and origin of the analyzed MPs were studied using a scanning electron microscope and energy-dispersive X-ray spectrometer (SEM/EDS), which offer high-resolution data on the surface condition and qualitative information on the chemical composition. In India, the SEM/EDS technique was used to characterize the MPs extracted from water^30,31,58,68^ and sediment.^18,30,31,58^ SEM/EDS is a time-consuming and expensive technology that is widely used to characterize the elemental composition and morphology. Moreover, chemical characterization may be vulnerable to selection bias, as the ability of the researcher determines how well the MPs are isolated.^21^

### 6.5 Atomic Force Microscope

Atomic force microscopy (AFM) is capable of producing images with nanometer resolutions, and its probes can be used to scan objects in both contact and noncontact modes. This technique is employed to examine the abrasion and weathering patterns such as flakes, cracks, pits, and adhering particles of MPs extracted from environmental matrices. With the AFM technique, it is possible to examine the morphological characteristics of MPs. For instance, Selvam et al.^66^ used AFM to study the morphological features of MPs extracted from ground and surface water in coastal south India. However, AFM has some limitations, including the need to scan samples at relatively modest rates to obtain high-quality images. Additionally, artifacts may be introduced due to interactions between the tip and the sample or image processing procedures.

## 7 Current Knowledge of MPs in a Freshwater Environment in India

### 7.1 Abundance and Distribution of MPs in Rivers

There is a significant fluctuation in the concentration of MPs detected in water and sediment samples obtained from freshwater environments in India. Figure 1 and Table 1 illustrate that more research on MPs has been conducted in northern and southern India, whereas there is a large void of data sets in the central parts of India. There is a flushing mechanism happening in the Northern rivers (perennial) compared to the Southern rivers (nonperennial). As a result, the rivers are behaving as temporary MPs sinks. Studies on water samples were mostly carried out in southern India, while studies on sediment samples were primarily conducted in northern India. Eight publications have discussed MPs in 13 rivers of different sizes. MPs were observed in both southern India (4 studies) and northern India (3 studies). The abundance and distribution of MPs displayed large variability, depending on the sampling methods utilized. For sediment samples, measurements were taken in items/kg, items/kgdw, and particles/kg, while for water samples, measurements were recorded in particles/L, items/m^3^, items/km^2^, and items/1000 m^3^.

**Figure 2:**
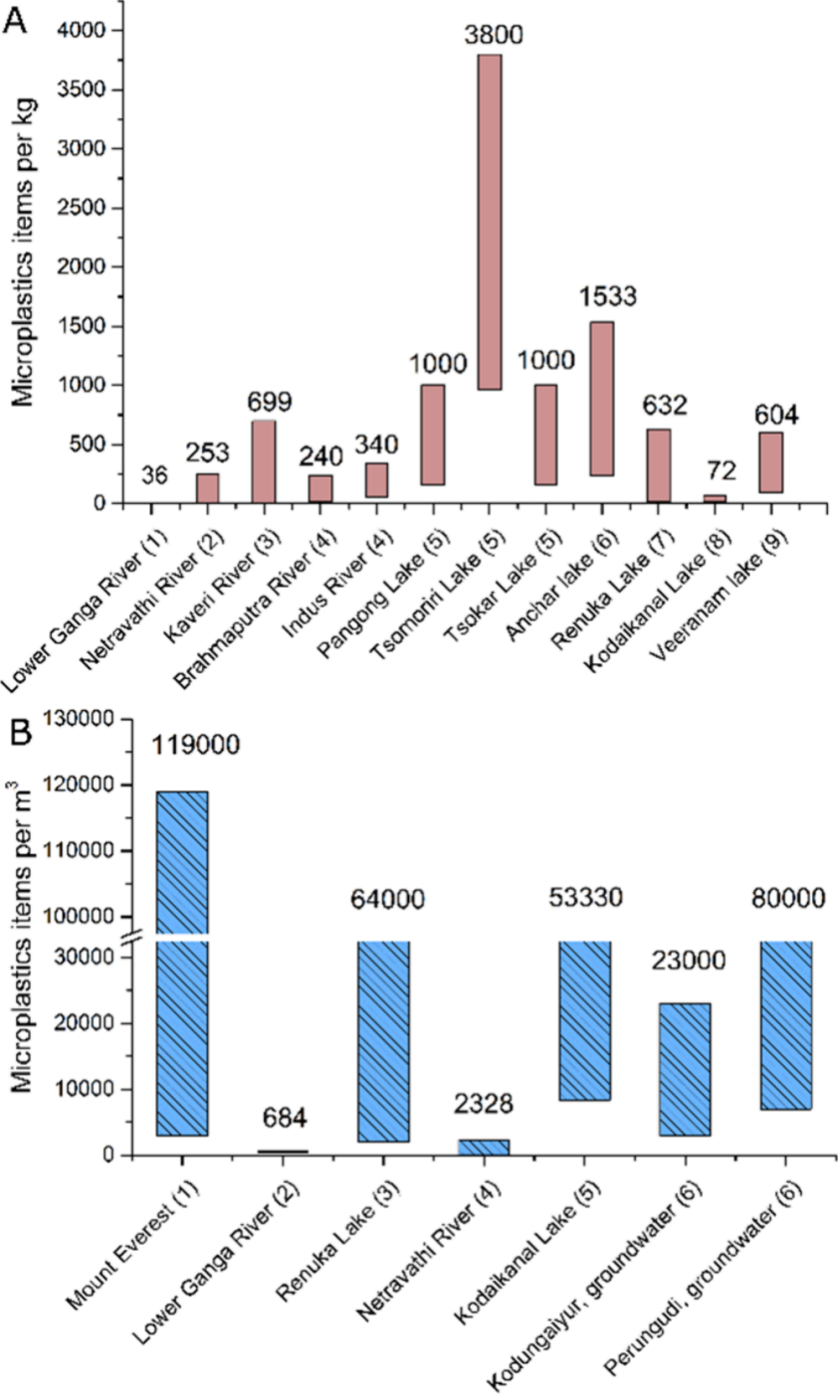
Concentrations of MPs from the freshwater systems of India. Sediment: 1. Singh et al.^57^; 2. Amrutha and Warrier^31^; 3. Maheswaran et al.^65^; 4. Tsering et al.^18^; 5. Tsering et al.^62^; 6. Neelavannan et al.^33^; 7. Ajay et al.^63^; 8. Laju et al.^59^; 9. Bharath et al.^60^ Water: 1. Napper et al.^70^; 2. Napper et al.^52^; 3. Ajay et al.^63^; 4. Amrutha and Warrier^31^; 5. Laju et al.^59^; 6. Bharath et al.^68^

Comparing data on MPs concentrations is challenging due to variations in measurement techniques. In southern India, the Kaveri (1–699 items/kg)^35^ and Netravathi River (9–253 items/kg)^31^ had higher concentrations of MPs in sediments compared to the lower Ganga River (17–36 items/kg),^57^ Indus (60–340 items/kg),^18^ and Brahmaputra (20–240 items/kg)^18^ (Figure 2A). In terms of river water samples, the Netravathi River had a lower MP concentration (288 items/m^3^) than the Ganga (466 items/m^3^),^52^ Adyar (330 items/m^3^),^32^ and Kosasthaiyar (670 items/m^3^)^32^ rivers (Figure 2B). India has the second-highest level of MPs pollution in its river systems after China when compared to other countries.^69^ Population density significantly influenced MPs concentration, as personal care product consumption, laundry wastewater amount, and human activity frequency all increase with population density. Northern Indian rivers originate in the Himalayas and pass through high-population-density urban areas, while southern Indian rivers do not flow continuously throughout the year and retain a significant number of MPs within sediments during low flow periods. This explains why MPs input into the ocean from rivers may be concentrated at different times of the year and is closely linked to the weather. River flow and MPs concentrations were found to be negatively correlated, with high flow diluting MPs concentration.^31,32,52,65^ Studies have reported that MPs abundance in the Ganga River was lower during the rainy season than in the dry season, likely due to the “flushing” mechanism of the river during the monsoon season. The density, buoyancy, and adsorption capacities of MPs can also influence their transport, migration, and distribution in surface water.^69^

### 7.2 Abundance and Distribution of MPs in Lakes

Less than 10 studies have reported on the distribution and abundance of MPs in lakes. The distribution of MPs in lake sediments has been investigated in 8 studies, with 3 studies conducted in south India and 5 studies conducted in northern parts of India. Similarly, 5 studies have examined the presence of MPs in lake water, with 4 studies conducted in south India and 1 study in northern parts of India. In the sediments of northern Indian lakes such as Anchar Lake (233–1533 items/kg), Pangong Lake (160–1000 items/kg dw), Tsomoriri Lake (960–3800 items/kg dw), Tsokar Lake (160–1000 items/kg dw), and Renuka Lake (180 ± 143 items/kg dw), the concentration of MPs is higher compared to southern Indian lakes such as Red Hills Lake (27 items/kg), Kodaikanal Lake (28.31 items/kg), and Veeranam lake (309 items/kg; Figure 2A).

It is worth noting that high-altitude Himalayan lakes exhibit a very high abundance of MPs. Some of the studied high-altitude Himalayan lakes includes Anchar Lake (1,500 m.a.m.s.l.), Pangong Lake (4,250 m.a.m.s.l.), Tsomoriri Lake (4,522 m.a.m.s.l.), and Tsokar Lake (4,572 m.a.m.s.l.; Figure 1). Among these lakes, the Anchar lake is located near a city, while Pangong and Tsomoriri lakes are endorheic and situated in remote areas. Tsering et al.^62^ found that the sources of MPs in these remote lakes were derived from rain and anthropogenic activities such as vehicles, tourism, tents, clothes, drinking water bottles, food packing, and plastic litter. Neelavannan et al.^33^ reported that Anchar Lake’s MPs have a complex source derived from the textile, packing, and automotive sectors.

In the southern parts of India, various studies have investigated the presence of MPs in different freshwater bodies. The concentration of MPs in Kodaikanal Lake water was found to be lower (24.42 items/L) than in Renuka Lake (21 items/L) and Red Hills Lake (5.9 items/L; Figure 2B). In Manipal Lake, the concentration of MPs was found to be higher during the monsoon season due to the surface runoff from surrounding areas and resuspension of MPs. Human activities have been identified as one of the most important factors contributing to the presence of MPs in freshwater bodies. In the coastal areas of South India, the concentration of MPs in groundwater was found to be higher along the Chennai coast (3–23 and 7–80 items/L) than in Tuticorin (10.1 items/L). Studies have also shown that the abundance of MPs in sediment cores around Kodaikanal Lake decreased with increasing depth, with the maximum concentration found in the top layers. This trend may be attributed to the increased use of plastic products in recent times. Most of the research on MPs in India has been conducted on sediments from the high-altitude Himalayan regions except for a few studies on water samples. Endorheic lakes have been identified as possible sinks for MPs due to their long-term water retention. Further research is needed to determine the sources of MPs, whether they are from the atmosphere or human activities, and to study their distribution in the environment.

### 7.3 Physical Properties of MPs

**Figure 3:**
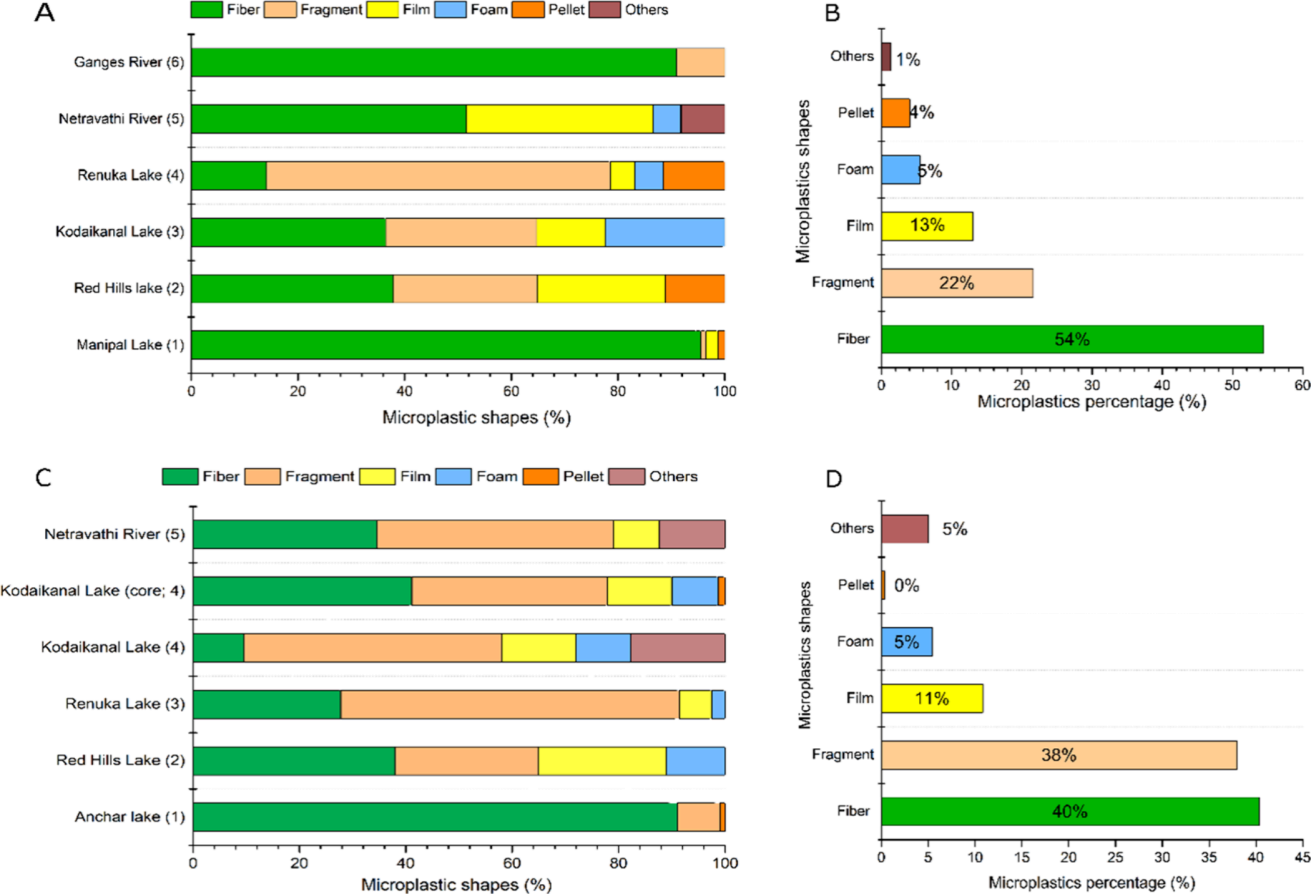
Shape-based proportions and relative abundance of MPs in freshwater systems of India. Water (A and B): 1. Warrier et al.^51^; 2. Gopinath et al.^30^; 3. Laju et al.^59^; 4. Ajay et al.^63^; 5. Amrutha and Warrier^31^; 6. Napper et al.^52^ Sediment (C and D): 1. Neelavannan et al.^33^; 2. Gopinath et al.^30^; 3. Ajay et al.^63^; 4. Laju et al.^59^; 5. Amrutha and Warrier.^31^

Understanding the shape of MPs in freshwater environments is crucial for assessing their fate and the potential impact on biota. Different shapes of MPs may behave differently in water bodies, with fibers and foams potentially floating and fragments possibly sinking to the bottom. The shape of MPs can also affect their impact on biota, with coarse fragments potentially damaging the digestive system of fish, while nanoparticles may translocate to organs. In India, studies have found a range of MPs shapes in freshwater environments, including fibers, films, fragments, pellets, and foams (Figure 3A, B). Fibers and fragments were the dominant shapes found in both north and south India (Figure 3B, D). For example, in Anchar Lake in the NW Himalayas, 91% of MPs were fibers. Similar observations were made in Manipal Lake (95% during monsoon and 96% postmonsoon) and the Ganga River (91%). Physical characterization of MPs also revealed that secondary MPs (fragments, fibers, films, and foams) were more common than primary MPs (pellets). Secondary MPs are typically formed from the fragmentation of larger plastic materials. The smaller MPs particles are harder to detect and also affect the result.

The sources of MPs can be determined by their shapes, where pellets are commonly traced back to cosmetic and industrial products, and fibers can be linked to fishing gear, synthetic clothing, and wastewater. Films are often derived from agricultural films and plastic bags, and foams can be attributed to packing materials and thermocol buoys. Studying the color of MPs can provide important insights into their properties, distribution, interactions with the environment, and potential impact on ecosystems and human health.^31^

Some studies have suggested that the color of MPs may affect how they are ingested by organisms.^33^ For example, brightly colored MPs may be more attractive to some organisms, which could increase their exposure to toxic chemicals associated with plastic particles.^31^ Gobies (ray-finned fish) are visual predators and are inclined to consume MPs that have colors similar to their prey.^75^ The color of plastics themselves will significantly affect how much sunlight is absorbed because different colors of plastics absorb light with varying wavelengths and energies.^76^

**Figure 4:**
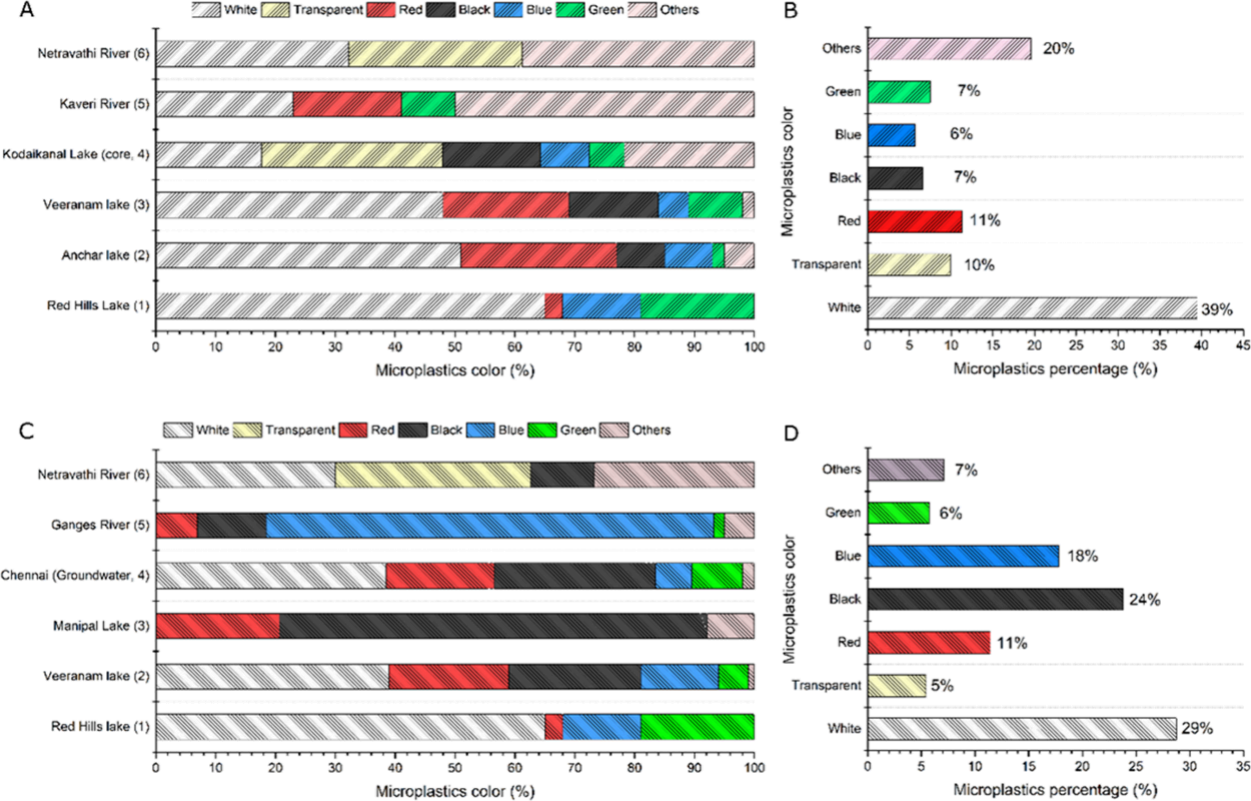
Proportions and relative abundance of MPs color present in freshwater ecosystems of India. Sediment (A and B): 1. Gopinath et al.^30^; 2. Neelavannan et al.^33^; 3. Bharath et al.^60^; 4. Laju et al.^59^; 5. Maheswaran et al.^65^; 6. Amrutha and Warrier.^31^ Water (C and D): 1. Gopinath et al.^30^; 2. Bharath et al.^60^; 3. Warrier et al.^51^; 4. Bharath et al.^68^; 5. Napper et al.^52^; 6. Amrutha and Warrier.^31^

Black or dark-colored MPs may absorb more heat from the sun than light-colored ones, which can accelerate the ice melt. The most common colors noticed in MPs of the freshwater environment were white. White MPs made up the majority (65%) of MPs in the sediment and water of Red Hills Lake.^30^ 51% of MPs detected in Anchar Lake bottom sediment were white in color.^33^ Colored MPs were also common.^52^ For example, multiple colors of MPs were noticed in the Veeranam Lake water sample, namely, red (20%), black (22%), blue (13%), green (5%), and yellow (1%).^60^ Colored MPs may have come from various sources including cloth wastes, fishing nets, ropes, and agricultural mulching applications.^31,62^ The white or transparent color MPs originate from carry bags and packaging materials.^30,33^ The major MPs color in the environment is summarized in Figure 4.

### 7.4 Polymer Types and Source of MPs

**Figure 5:**
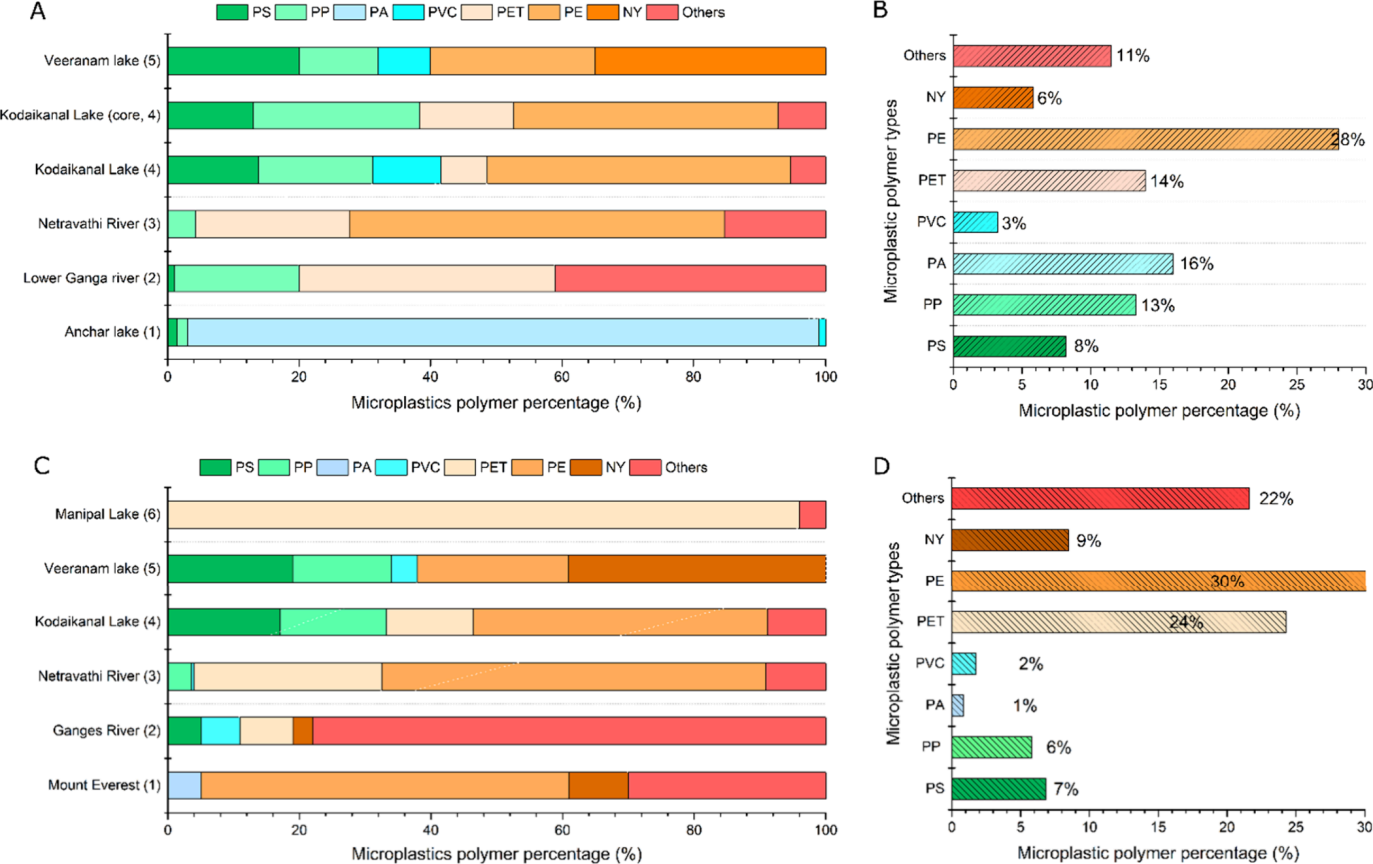
Polymer-based proportions and relative abundance of MPs in freshwater ecosystems of India. Sediment (A and B): 1. Neelavannan et al.^33^; 2. Singh et al.^57^; 3. Amrutha and Warrier^31^; 4. Laju et al.^59^; 5. Laju et al.^59^; 6. Bharath et al.^60^ Water (C and D): 1. Napper et al.^70^; 2. Napper et al.^52^; 3. Amrutha and Warrier^31^; 4. Laju et al.^59^; 5. Bharath et al.^60^; 6. Warrier et al.^51^

Most of the studies reviewed employed spectroscopic techniques to confirm the polymer types of MPs in freshwater environments. Polypropylene (PP), poly(ethylene terephthalate) (PET), and polyethylene (PE) were found to be the most common polymer types, accounting for 74% of global plastic production in 2015. These materials are commonly used in short-life-cycle products. Other polymers, such as polyamide (PA), poly(vinyl chloride) (PVC), polystyrene (PS), nylon (NY), and cellulose (CL), were also detected in some studies (Figure 5). The rate of weathering of MPs is influenced by biofouling, exposure to UV light from the sun, and hydrodynamic conditions. The physical characteristics and surface morphology of MPs were investigated through visual inspection and scanning electron microscopy (SEM), which allows for high-resolution visualization of the morphological cracks of an object and can provide information to determine the weathering stages of MPs.

The carbonyl index (CI) of MPs is analyzed using FTIR spectroscopy to assess their aging and weathering patterns. However, no research has been conducted in India on the weathering patterns of MPs in freshwater environments based on their carbonyl index values. While advanced analytical techniques such as Raman and FTIR spectroscopy can identify the polymer types of MPs, most studies in India and other countries rely on visual identification, which may introduce bias due to the analyst’s expertise, sample matrix, particle size, and shape. To confirm the polymer types of MPs, it is recommended to use spectroscopic instruments or other analytical methods in addition to visual inspection, especially for small particles.

At present, there is no clear understanding of the factors that contribute to the variability of polymer types in freshwater environments, and further research is needed to determine if there are specific groups of polymers that are more prevalent in MPs in different locations and over different distances traveled.^71^ However, it is known that two of the most commonly found polymers in freshwater environments, PP and PE, have a density lower than that of water, which means that they can be widely dispersed in the water and taken up by aquatic organisms.^77^ Over time, as biofouling occurs, the density of these polymers may increase, causing them to sink and become deposited in sediment.^33,51^ As PET has a density of 1.39 g/cm^3^, it will easily pass through the freshwater column and become mixed with the sediment. The denser PET fibers sink and get mixed with the sediments, particularly during the postmonsoon season as the water level drops and the turbulence intensity reduces. Warrier et al.^51^ studied the MPs seasonal variation in Manipal Lake and found that polymer types are a function of seasonality, while PET dominates (∼96%) the postmonsoon season in Manipal Lake. The movement and distribution of MPs in freshwater environments are influenced by a range of hydrodynamic factors such as wind, current, and wave patterns.

### 7.5 Future Outlook

Over the past decade, there has been an increase in scientific interest in MPs research work, leading to an expansion of knowledge. However, there are still important concerns and problems that require attention. We assessed the entire central parts of India as a “white spot”, indicating the presence of very limited field data. Since central parts of India are heavily populated, this is a concern as central parts of India include many large river systems such as the Mahanadi, Godavari, and Krishna. Such data would not only help quantify the extent of MPs contamination in Indian river systems but also help to better understand the land-to-ocean MPs fluxes and the role these large Indian rivers play to control MPs in the marine ecosystem. The other important observation was the presence of MPs in some of the most pristine lakes in the Himalayas (e.g., Pangong and Tsomoriri Lake). However, data sets are very limited, and more studies are required in the Himalayan region to constrain the impact of MPs on the Himalayan ecosystem already fragilized by climate change. We would like to emphasize that despite the reported ability of plastic particles to be carried by wind^73^ and reach even remote glaciers^74^ no research has been conducted on the impact of MPs on the Himalayan cryosphere.

The other and perhaps more important observation was irregularities in the sampling setup and MPs characterization work. We invoke the fact that it is important to develop unified and integrated sampling and processing techniques for all future research work. The literature review reveals that net sampling is considered the most effective method for quantifying MPs levels in water samples. This method offers several advantages, such as covering a large sampling area and reducing the water volume of samples, which save time. However, we found it difficult to compare results across studies as different net aperture sizes, trawling speeds, and sampling durations were used. These are important parameters and must be uniformly followed. For example, the net aperture size plays a critical role in determining MPs abundance, as smaller mesh size results in higher MPs abundance. Therefore, to ensure accurate and consistent results, we recommend to use a 333 μm mesh size when trawling for 30 min at a speed of 3–4 knots during surface water sampling. If the water has a lot of floating vegetation or plant life, a pump and filtration method can be used as an alternative sampling technique.^72^ To measure the level of MPs in sediments, the abundance of MPs remained consistent regardless of the type of sampling tool used. Therefore, both grab samplers (bottom or bed load) and metal spoons/shovels (shoreline) are appropriate for collecting sediments from lakes and rivers. These sampling methods allow for the collection of a substantial amount of sediment, which is necessary for obtaining meaningful MPs concentrations.^72^ It is important to note that airborne fibers can significantly overestimate MPs in all environmental matrices, including water and sediment. Therefore, it is crucial to check for background MPs contamination during both the sampling and laboratory processes. The amount of MPs found in the environment can also be significantly influenced by the pore size of the filter. Cai et al.^78^ demonstrated the laboratory experiment, and field validation showed that a membrane filter with smaller pores (<20 μm) could retain more particles. Therefore, we recommend using a filter with a 20 μm pore size membrane.

The MPs properties of the particles are affected by both transport and depositional processes in the aquatic environments. When compared to sediment mobility, the transport behavior of MPs is different in terms of particle density, form, and consequences like biofouling. The laboratory experiments by Kowalski et al.^79^ demonstrated that the settling velocity of MPs is mostly influenced by their shape. Regarding the exchange processes in the water column, the contact with the river bed, and the features of current turbulence, the transport of MPs with higher densities and a similar particle size is equivalent to sediments.^80^ Therefore, the sampling of MPs particles is significantly influenced by the local circumstances (as mentioned above) as well as by the sampling seasons. The following distinguishes between the location and compartment (sediment and water). The water and sediment can be sampled as volume-reduced or bulk samples depending on the research questions.^81^ The water surface and the water column are the two components of the water compartment. Sediments can be recovered from the bottom, the shore, or the alluvial plains of the river, depending on areas of accumulation or remobilization. Additionally, samples may be disturbed by bioturbation, so sampling depth must be taken into account.^82^

Sites are separated into lakes and rivers (with alluvial plains). Rivers are intricate systems because the morphology of the surface water affects how MPs are deposited. For example, the depth, width, transect shape, sinuosity, bottom gradient, braiding, level of anastomosis, and vegetation along the river banks control the MPs deposition.^83^ Additionally, if rivers do not flow naturally, consideration must be given to the deposition of plastics in the regulated portion (such as groynes, barrages, and dams). In addition to the river mouth, samples may be taken from the water’s surface and column, above the ground, in the river’s channel, nearby the beach, from the cut bank or point bar, or from the river’s channel itself.^82^

Standardization is required for the extraction of MPs from environmental samples, much like the standardization needed for sampling strategies. MPs are usually separated using a density separation technique involving a sodium chloride (NaCl) solution. Sodium chloride (NaCl) solution is commonly used in density separation methods due to its affordability and environmental friendliness. However, it underestimates MPs with densities higher than 1.2 g cm^–3^. To address this limitation, the use of a sodium iodide (NaI) solution is recommended for the density separation process instead of a NaCl solution. NaI solution provides better separation of high-density MPs, making it a more effective alternative for the density separation process. We therefore urge the MPs research community to adapt a uniform sampling strategy and characterization work. Further, researchers must follow strict contamination control procedures during sampling and laboratory analysis to obtain reliable MPs data. The following control measures should be considered during MPs analysis: (i) covering all materials and solutions with glass lids or aluminum foil, (ii) filtering and storing all solutions in glass containers, (iii) using procedural blanks, field blanks, and open filters to control the deposition of MPs from the air, (iv) avoiding plastic tools, (v) wearing a cotton lab coat and using a cleaned laminar flow hood, and (vi) using high-quality glass fiber filters.^55^

## 8 Conclusions

MPs are a growing concern as emerging contaminants in aquatic environments worldwide. In recent years, the presence and distribution of MPs in rivers and lakes have gained attention from researchers, policymakers, and the general public. However, our current understanding of the abundance, distribution, and sources of MPs in freshwater ecosystems remains limited. In India, studies of MPs in freshwater ecosystems have mainly been conducted over the past decade. However, comparing the concentration levels of MPs in different environmental matrices is challenging due to variations in sample collection, processing, and analytical procedures. Furthermore, many of the studies conducted in India do not provide details on quality assurance and quality control. To address some of these challenges and improve our understanding of MPs, we propose standardized definitions for MPs size and monitoring techniques for water and sediment. Further, the baseline level of MPs in major Indian rivers has not been studied. Despite significant progress in global toxicological studies of MPs, further research is needed to better understand the abundance, sources, and pathways of MPs, particularly in the central parts of India and high-altitude Himalayan Mountain regions.
